# An Efficient Synthetic Approach Towards Benzo[*b*]pyrano[2,3-*e*][1,4]diazepines, and Their Cytotoxic Activity

**DOI:** 10.3390/molecules25092051

**Published:** 2020-04-28

**Authors:** Islam H. El Azab, Nadia A.A. Elkanzi

**Affiliations:** 1 Chemistry Department, Faculty of Science, Taif University, Al-Haweiah, P.O. Box 888, Taif 21974, Saudi Arabia; 2 On leave from Chemistry Department, Faculty of Science, Aswan University, Aswan, P.O. Box, Aswan 81528, Egypt; kanzi20@yahoo.com; 3 Chemistry Department, College of Science, Jouf University, P.O. Box 2014, Sakaka, Saudi Arabia

**Keywords:** cyclocondensation reaction, benzodiazepine, *N*-heterocycles, cytotoxic activity

## Abstract

In search of unprecedented tri and/or tetrapod pharmacophoric conjugates, a series of 32 new 4-ethyl-1*H*-benzo[*b*][1,4]diazepin-2(3*H*)-ones were synthesized and properly elucidated using MS, IR, NMR, and elemental analysis. *In vitro* investigation of 11 compounds of this series, using a panel of two human tumor cell lines namely; human breast adenocarcinoma (MCF-7), and human colorectal carcinoma (HCT-116), revealed promising cytotoxic activities. Among all synthesized compounds, analogue **9** displayed maximum cytotoxicity with IC_50_ values of 16.19 ± 1.35 and 17.16 ± 1.54 μM against HCT-116 and MCF-7, respectively, compared to standard drug doxorubicin.

## 1. Introduction

**Figure 1:**
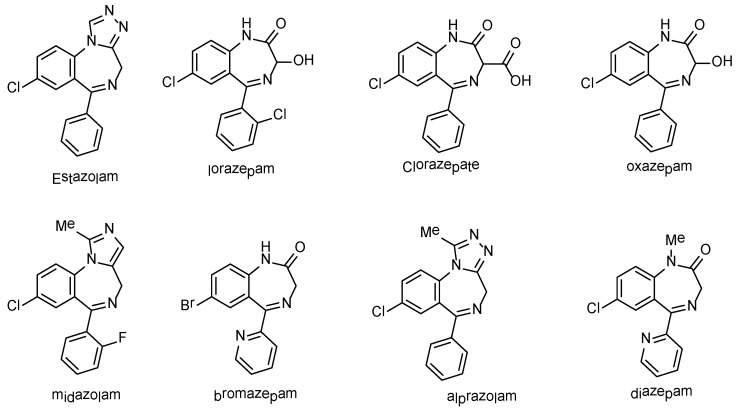
Representative examples of biologically active 1,4-benzodiazepines.

Benzodiazepines (BDZ’s) are privileged heteroaromatic molecules and were considered to be the core of an essential class of pharmaceutically active analogues, so, their synthesis is of high value in the subject of medicinal and pharmaceutical chemistry [1,2,3,4]. 1,4-Benzodiazepines (Figure 1) are used as anti-microbial [5], in alcohol withdrawal syndrome (AWS) [6], as endothelin antagonist [7], hypnotics [8], anxiolytics [9], anticonvulsants [10], muscle relaxants [10], anticancer drugs [11,12], sedatives [13,14], and antipsychotics [15]. Recently, benzodiazepines were also recognized to have anti-proliferative [16], antimicrobial [17], anti-inflammatory [18], anti-plateletanti-ulcer [19], and analgesic [20].

Due to our admiration with the synthesis, modification, and studying the biological activities of benzodiazepines, herein, we freshened our sustained efforts [21,22,23,24,25,26,27], through the synthesis and utility of 5-methyl-4-(methylthio)-2-oxo-2,11-dihydrobenzo[*b*]pyrano[2,3-*e*][1,4]diazepine-3-carbonitrile (**5**) as a reactive precursor, for the annulation of benzopyranodiazepines of potential biological activity.

## 2. Results and Discussion

### 2.1. Chemistry

**Scheme 1:**
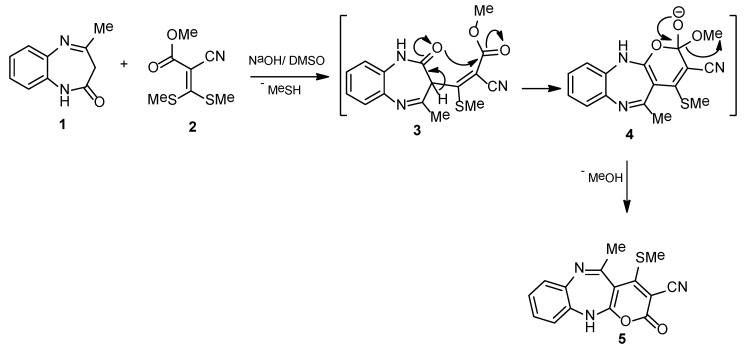
Synthesis of benzo[*b*]pyrano[2,3-*e*][1,4]diazepine derivative **5**.

In our sustained efforts to synthesize various functionalized heterocyclic analogues, and studying their biological activities [21,22,23,24,25,26,27], we desired to report a new efficient and simple technique for the synthesis of benzo[*b*]pyrano[2,3-*e*][1,4]diazepines. Compound **1** [28] was selected as a substrate to condense with compound **2** [29] in stirred DMSO, containing catalytic amounts of NaOH at rt to furnish pyrano[1,4]diazepine derivative **5** with an 85% yield (Scheme 1). The existence of the nitrile group was assured using its IR absorption band at 2209 cm^−1^ and its ^13^C NMR as a singlet at 115.9 ppm, while the occurrence of the methylthio moiety was supported using ^1^H NMR as a singlet at 2.73 ppm. Further, evidence for compound **5** was obtained from its noted mass at *m*/*z* 297.06 matches with the formula C_15_H_11_N_3_O_2_S.

**Scheme 2:**
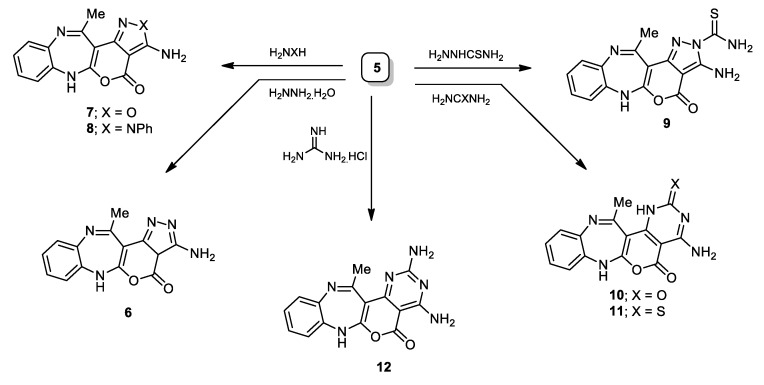
Synthetic pathways of compounds **6**–**12**.

The good replaceable active methylthio moiety in compound **5** was cyclized with NH_2_NH_2_∙H_2_O, afforded the amino pyrazole derivative **6** with a 65% yield (Scheme 2). Similarly, it was smoothly condensed with hydroxylamine, phenyl hydrazine, thiosemicarbazide, urea, thiourea, and guanidine hydrochloride to provide the target compounds (**7**–**12**). The gesture of the methylthio protons initially observed in compound **5** (^1^H NMR) at 2.73 ppm was vanished, whereas the NH_2_ protons were observed around *δ* ~ 7.00 ppm.

**Scheme 3:**
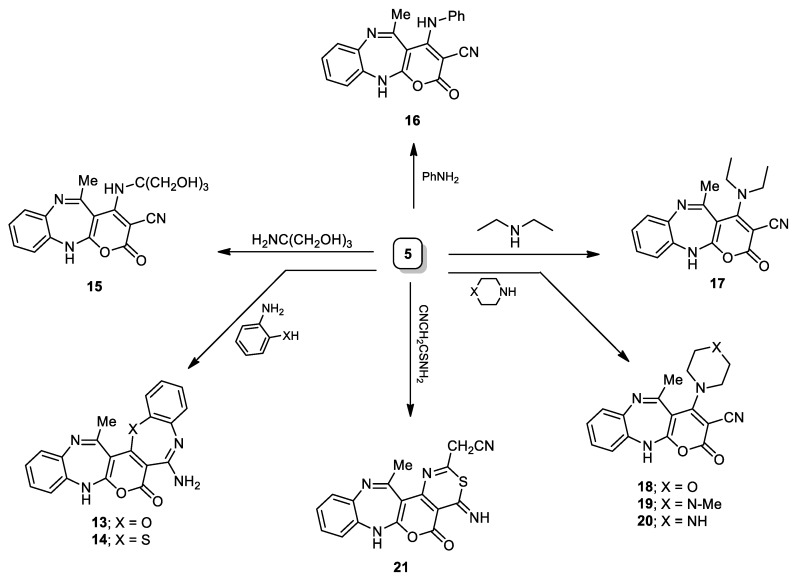
Synthetic pathways of compounds **13**–**21**.

Moreover, compound **5** was condensed with 2-aminophenol or 2-aminothiophenol, and benzo[*b*][1,4]oxa(thia)zepine analogues (**13** and **14**) were obtained (Scheme 3). The construction of compound **13** was presumed to advance *via* the preliminary condensation of the hydroxyl proton of 2-aminophenol and the methylthio moiety of compound **5**, through removal of the methanethiol fragment, followed by further, internal cyclization to oxazepine derivative **13**, through nucleophilic attack of the amino moiety onto the nitrile. The noted mass at *m*/*z* 358.11 matching with the formula C_20_H_14_N_4_O_3_, as well the NH_2_ band (IR) at ~3325 cm^−1^, and its broad singlet (^1^H NMR) at 8.61 ppm, all confirmed structure **13**. Upon treatment of compound **5** with tris(hydroxymethyl)aminomethane or PhNH_2_, the secondary amine analogues (**15** and **16**) were obtained. Compound **15** (IR) showed interest bands centered at 2217 and 3406 cm^−1^, owing to the hydroxyl and the nitrile groups, correspondingly, while, its ^1^H NMR scope exhibited three new singlets at 3.02, 3.81, and 5.74 ppm, owing to the hydroxyl, methylene, and the secondary amine protons, respectively. In a similar manner, compound **5** was subjected to some selected secondary amines namely; diethylamine, morpholine, *N*-methylpiperazine and/or piperazinyl to furnish the corresponding tertiary amines (**17**–**20**). Strong absorption sign centered around 2210 cm^−1^ owing to the nitrile group, supported these structures. Compound **17** (^1^H NMR) demonstrated a triplet and quartet signs allocated to the two equivalent ethyl moieties at 1.16 and 3.35 ppm, respectively. Furthermore, compound **5** was cyclocondensed with cyanothioacetamide, afforded 1,3-thiazine analogue **21** with an 80% yield (Scheme 3). The cyanomethyl and imino protons in compound **21** appeared as two singlets (^1^H NMR) at 4.15 and 8.91 ppm, respectively.

**Scheme 4:**
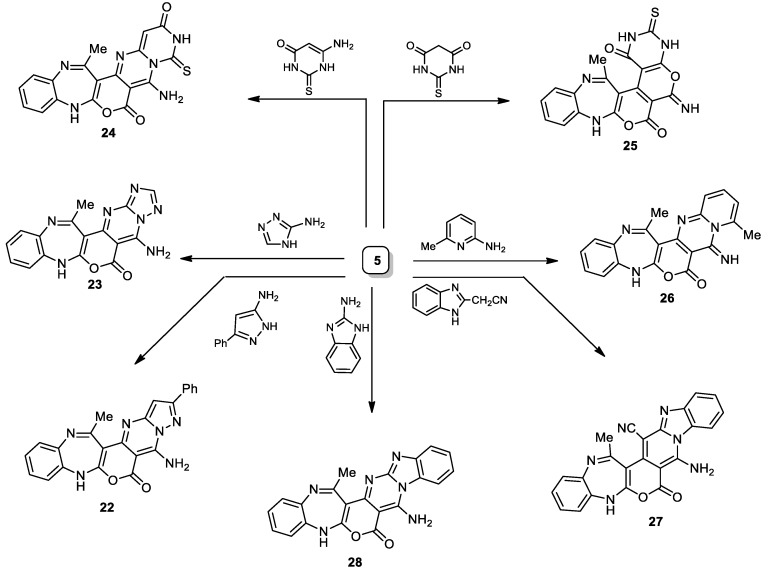
Synthetic pathways of compounds **22**–**28**.

Similarly, upon smooth cyclocondensation of compound **5** with some heterocyclic amines, a series of ploy fused heterocyclic systems (**22**–**28**) were acquired (Scheme 4).

**Scheme 5:**
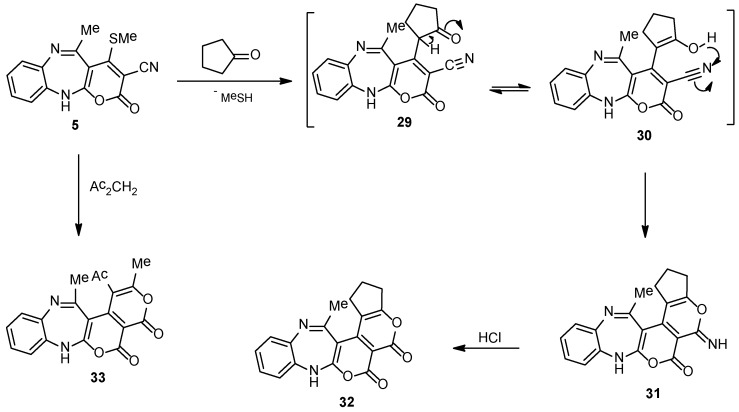
Synthetic pathways of compounds **31**–**33**.

The adaptability of our heteroaromatic construction policy was spare, as demonstrated by the annulation of pyrano[*c*]pyran analogue. Thus, cyclocondensation of intermediate **5** with cyclopentanone or acetylacetone, afforded the pyrano[*c*]pyran derivatives (**32** and **33**), respectively (Scheme 5). The formation of compound **31** could be supposed to advance through the preliminary condensation of the energetic methylene in the cyclopentanone with the easily removable methylthio moiety in compound **5**, to afford the non-isolable intermediates **29** and **30**, which underwent internal cyclization through a nucleophilic attack of the OH to the C≡N fragment, to furnish the final product **31**. The exocyclic imino moiety in compound **31**, was easily converted to carbonyl via treatment with HCl in boiling EtOH, to afford cyclopenta[*b*]pyran-2-one derivative **32** (Scheme 5). Compound **32** (IR) showed intense bands at 1705, 3256 cm^−1^ owing to the carbonyl and imino moieties, correspondingly, whereas its mass spectrum presented a molecular ion peak (C_19_H_14_N_2_O_4_) at *m*/*z* 334.10.

**Scheme 6:**
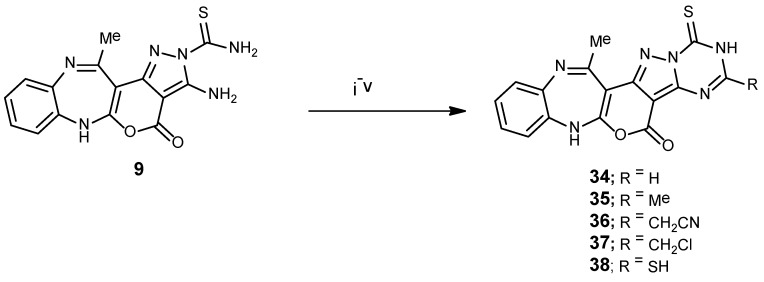
Reagents and conditions for synthesis of compounds **34**–**38**. Note: i = HC(OEt)_3_; ii = AcCl; iii = CNCH_2_CO_2_Et; and iv = ClCOCH_2_Cl; v = CS_2_/EtOH.

Finally, α-aminocarbothiamide analogue **9** was applied as a facile point to construct the target pyrazolo[1,5-*a*][1,3,5]triazine-4-thiones (**34**–**38**) (Scheme 6), through smooth condensation with triethyl orthoformate, acetyl chloride, ethyl cyanoacetate, chloroacetyl chloride, and carbon disulfide. The mass spectrum of compound **36** presented an ion peak at *m*/*z* 389.05 (C_19_H_14_N_2_O_4_), whereas its IR declared two distinctive bands owing to the C=S and C≡N moieties, at 1325 and 2215 cm^−1^ correspondingly.

### 2.2. Pharmacological Evaluation

#### Cytotoxic Impact

According to the Sulforhodamine B (SRB) method [30,31], new eleven conjugates were *in vitro* examined for their cytotoxic impact towards human colorectal carcinoma (HCT-116) and human breast adenocarcinoma (MCF-7). Doxorubicin was the positive drug, while DMSO was the negative control. The cytotoxic impacts of the investigated compounds are presented in Table 1.

Analyses of the IC_50_ data as given in Table 1 declare that, majority of the investigated compounds own noteworthy cytotoxic activities versus these cell lines. Where, compound **9** was the most effective among the screened series with IC_50_ = 16.19 ± 1.35 and 17.16 ± 1.54 μM against HCT-116 and MCF-7, respectively. Moreover, the test cell lines were generally susceptible to conjugates **7**, **6**, **8**, **15**, **16**, **18**, **19**, and **20**, in a downward order, with IC_50_ < 60.00 μM. Extra reading of the obtained results stated that, compounds **5** and **17** were less robust towards the two tumor cell lines with IC_50_ > 60.00 μM.

In conclusion, a series of 32 new benzo[*b*][1,4]diazepines was synthesized as a bipod or tripod pharmacophoric architectures that could reinforce the cytotoxic impact. The presence of the parent skeleton benzo-pyrano-diazepine was vital for the extensive spectrum of cytotoxic action against the screened cell lines. Moreover, introducing the pyrazole ring bearing carbothioamide fragment to the parent skeleton enhanced the anti-tumor ability of compound **9** to become close to the doxorubicin. The cytotoxic activity of compounds **7**, **6**, and **8** was attributed to the presence of the amino oxazole as well as amino pyrazole moieties, in conjugation with the parent benzo-pyrano-diazepine skeleton. Whereas introduction of 1,1,1-tri hydroxymethyl methyl amine moiety in compound **15**, enforced it to show an excellent potency; moreover, the existence of a phenylamino fragment in compound **16** improved the molecule to show a strong cytotoxicity. On the other hand, introducing the piperazinyl or morpholinyl moiety in compounds **17**–**20** minified the potency of these molecules, compared to the other tested compounds.

## 3. Materials and Methods

### 3.1. General Information

Reagents were purchased from Sigma Aldrich (Bayouni Trading Co. Ltd., Al-Khobar, Saudi Arabia) and used without further purification. The reaction progress was monitored by TLC on silica gel pre-coated F254 Merck plates (Merck, Darmstadt, Germany). Spots were visualized by ultraviolet irradiation. Melting points were determined on a digital Gallen-Kamp MFB-595 instrument (Gallenkamp, London, UK) using open capillary tubes and were uncorrected. IR spectra were recorded as potassium bromide discs using Bruker-Vector 22 FTIR spectrophotometer (Bruker, Manasquan, NJ, USA). The NMR spectra were recorded with a Varian Mercury VXR-300 NMR spectrometer (Bruker, Marietta, GA, USA) at 300 and 75 MHz for ^1^H and ^13^C NMR spectra, respectively, using DMSO-*d*_6_ as the solvent. Mass spectra were recorded on a Hewlett Packard MS-5988 spectrometer (Hewlett Packard, Palo Alto, CA, USA) at 70 eV. Elemental analyses were conducted at the Micro-Analytical Center of Taif University, Taif, KSA. The pharmaceutical activity assays were carried out at the applied research sector, Egyptian company for vaccine and serum (VACSERA, Cairo, Egypt).

*5-Methyl-4-(methylthio)-2-oxo-2,11-dihydrobenzo[b]pyrano[2,3-e][1,4]diazepine-3-carbonitrile* (**5**). Compound **1** [28] (0.17 g, 1 mmol), methyl 2-cyano-3,3-dimethylthioacrylate [29] (0.20 mg, 1 mmol) and powdered KOH (0.08 g, 1.5 mmol) in DMSO (25 mL), were stirred at rt for 3 h. The reaction mixture was transferred onto mashed ice under energetic stirring for 1 h. The isolated product was collected, dried, and recrystallized using EtOH to furnish **5** as a pale yellow solid, with an 85% yield; mp 149–151 °C; IR (KBr): (cm^−1^) 1612 (C=N_*str*._), 1705 (C=O_*str*._), 2209 (C≡N_*str*._), 3206 (N-H_*str*._); ^1^H NMR (300 MHz, DMSO-*d*_6_): δ 2.01 (s, 3H, Me), 2.73 (s, 3H, -SMe), 6.80–7.21 (m, 5H, Ar-H and N-H_Diazep._); ^13^C NMR (75 MHz, DMSO-*d*_6_): 15.3, 25.6 (2Me), 115.9 (C≡N), 76.8, 86.4, 113.6, 123.5, 124.1, 126.6, 128.5, 137.3, 138.1 (9 C=C), 149.5 (C=O), 164.5 (C=N), 179.8 (=C-S-); MS (*m*/*z*, %): 297.06 (M^+^, 35); Anal. Calcd. for C_15_H_11_N_3_O_2_S (297.33): C, 60.59; H, 3.73; N, 14.13%. Found: C, 60.21; H, 3.35; N, 14.01%.

General procedure of the synthesis of compounds (**6**–**28**). Compound **5** (0.29 g, 1 mmol) and some selected amino compounds namely; ethylenediamine, hydrazine hydrate, hydroxylamine hydrochloride, phenyl hydrazine, thiosemicarbazide, urea, thiourea, guanidine hydrochloride, 2-aminophenol, 2-aminothiophenol, 2-amino-2-(hydroxymethyl)propane-1,3-diol, aniline, secondary amines, 2-cyanothioacetamide, 5-amino-3-phenyl-1*H*-pyrazole, 6-amino-2-thioxo-2,3-dihydropyrimidin-4(1*H*)-one, 3-amino-1,2,4-triazole, 2-thioxodihydropyrimidine-4,6(1*H*,5*H*)-dione, 6-methylpyridin-2-amine, 2-cyanomethyl benzimidazole, and 2-amino benzimidazole in 25 mL of EtOH has Et_3_N (0.3 mL), was refluxed for 7–10 h and the reaction advance was checked by TLC. After evaporation of EtOH, the crude product was triturated with acetone, and the isolated solid was filtered and purified by crystallization, using the proper solvent to furnish compounds (**6**–**28**) in fair yields.

*3-Amino-12-methyl-1H-benzo[b]pyrazolo[3′,4′:4,5]pyrano[2,3-e][1,4]diazepin-4(6H)-one* (**6**). Yellow powder (EtOH) with a 65% yield; mp 205–207 °C; IR (KBr): (cm^−1^) 1610–1623 (3C=N_*str*._), 1705 (C=O_*str*._), 3206–3325 (N-H_*str*._, N-H_2*str*._); ^1^H NMR (300 MHz, DMSO-*d*_6_): δ 2.01 (s, 3H, Me), 2.51 (s, 1H, Pyran_(C3)_-H), 6.80–7.21 (m, 5H, Ar-H and N-H_Diazep._), 8.43 (brs, 2H, NH_2_, D_2_O-exchangeable); MS (*m*/*z*, %): 281.09 (M^+^, 15); Anal. Calcd. for C_14_H_11_N_5_O_2_ (281.27): C, 59.78; H, 3.94; N, 24.90%. Found: C, 59.47; H, 3.63; N, 24.59%.

*3-Amino-12-methylbenzo[b]isoxazolo[3′,4′:4,5]pyrano[2,3-e][1,4]diazepin-4(6H)-one* (**7**). Yellow crystal (EtOH) with a 71% yield; mp 241–243 °C; IR (KBr): (cm^−1^) 1610–1623 (2C=N_*str*._), 1705 (C=O_*str*._), 3206–3325 (N-H_*str*._, N-H_2*str*._); ^1^H NMR (300 MHz, DMSO-*d*_6_): δ 2.01 (s, 3H, Me), 6.80–7.21 (m, 7H, Ar-H, N-H_Diazep__._ and NH_2_); MS (*m*/*z*, %): 282.07 (M^+^, 50); Anal. Calcd. for C_14_H_10_N_4_O_3_ (282.25): C, 59.57; H, 3.57; N, 19.85%. Found: C, 59.31; H, 3.20; N, 19.52%.

*3-Amino-12-methyl-2-phenyl-2H-benzo[b]pyrazolo[3′,4′:4,5]pyrano[2,3-e][1,4]diazepin-4(6H)-one* (**8**). Yellow solid (MeOH) with a 65% yield; mp 223–225 °C; IR (KBr): (cm^−1^) 1610–1623 (2C=N_*str*._), 1705 (C=O_*str*._), 3206–3325 (N-H_*str*._, N-H_2*str*._); ^1^H NMR (300 MHz, DMSO-*d*_6_): δ 2.01 (s, 3H, Me), 6.80–7.21 (m, 13H, Ar-H, N-H_Diazep__._ and NH_2_); MS (*m*/*z*, %): 357.12 (M^+^, 15); Anal. Calcd. for C_20_H_15_N_5_O_2_ (357.37): C, 67.22; H, 4.23; N, 19.60%. Found: C, 67.12; H, 4.10; N, 19.42%.

*3-Amino-12-methyl-4-oxo-4,6-dihydro-2H-benzo[b]pyrazolo[3′,4′:4,5]pyrano[2,3-e][1,4]diazepine-2-carbothioamide* (**9**). Pale-yellow powder (EtOH) with a 75% yield; mp 143–145 °C; IR (KBr): (cm^−1^) 1325 (C=S_*str*._), 1610–1623 (2C=N_*str*._), 1705 (C=O_*str*._), 3206–3325 (N-H_*str*._, N-H_2*str*._); ^1^H NMR (300 MHz, DMSO-*d*_6_): δ 2.01 (s, 3H, Me), 6.80–7.21 (m, 7H, Ar-H, N-H_Diazep__._ and NH_2_), 8.53 (brs, 2H, CSNH_2_, D_2_O-exchangeable); MS (*m*/*z*, %): 340.07 (M^+^, 25); Anal. Calcd. for C_15_H_12_N_6_O_2_S (340.36): C, 52.93; H, 3.55; N, 24.69%. Found: C, 52.71; H, 3.26; N, 24.35%.

*4-Amino-13-methylbenzo[b]pyrimido[4′,5′:4,5]pyrano[2,3-e][1,4]diazepine-2,5(1H,7H)-dione* (**10**). Yellowish-brown solid (EtOH) with an 82% yield; mp 165–167 °C; IR (KBr): (cm^−1^) 1327 (C=S_*str*._), 1610–1623 (2C=N_*str*._), 1705–1715 (2C=O_*str*._), 3206–3325 (N-H_*str*._, N-H_2*str*._); ^1^H NMR (300 MHz, DMSO-*d*_6_): δ 2.01 (s, 3H, Me), 6.80–7.21 (m, 5H, Ar-H and N-H_Diazep__._), 8.61 (brs, 2H, NH_2_, D_2_O-exchangeable), 12.51 (brs, H, NH_Pyrim_., D_2_O-exchangeable); ^13^C NMR (75 MHz, DMSO-*d*_6_): 25.6 (Me), 76.8, 91.5, 113.6, 123.5, 124.1, 126.6, 128.5, 137.3, 138.1, 152.5 (9 C=C), 156.2, 162.7 (2C=O), 164.5, 167.1 (2C=N); MS (*m*/*z*, %): 310.01 (M^+^+1, 40); Anal. Calcd. for C_15_H_11_N_5_O_3_ (309.28): C, 58.25; H, 3.58; N, 22.64%. Found: C, 58.04; H, 3.21; N, 22.34%.

*4-Amino-13-methyl-2-thioxo-1,2-dihydrobenzo[b]pyrimido[4′,5′:4,5]pyrano[2,3-e][1,4]diazepin-5(7H)-one* (**11**). Yellow crystals (MeOH) with an 80% yield; mp 205–207 °C; IR (KBr): (cm^−1^) 1321 (C=S_*str*._), 1610–1623 (2C=N_*str*._), 1705 (C=O_*str*._), 3206-3325 (N-H_*str*._, N-H_2*str*._); ^1^H NMR (300 MHz, DMSO-*d*_6_): δ 2.01 (s, 3H, Me), 6.80–7.21 (m, 5H, Ar-H and N-H_Diazep__._), 8.61 (brs, 2H, NH_2_, D_2_O-exchangeable), 10.32 (brs, H, NH_Pyrim_., D_2_O-exchangeable); MS (*m*/*z*, %): 325.06 (M^+^, 55); Anal. Calcd. for C_15_H_11_N_5_O_2_S (325.35): C, 55.38; H, 3.41; N, 21.53%. Found: C, 55.02; H, 3.15; N, 21.24%.

*2,4-Diamino-13-methylbenzo[b]pyrimido[4′,5′:4,5]pyrano[2,3-e][1,4]diazepin-5(7H)-one* (**12**). Yellow crystals (EtOH) with a 75% yield; mp 221–223 °C; IR (KBr): (cm^−1^) 1610–1623 (2C=N_*str*._), 1705 (C=O_*str*._), 3206–3325 (N-H_*str*._, 2N-H_2*str*._); ^1^H NMR (300 MHz, DMSO-*d*_6_): δ 2.01 (s, 3H, Me), 6.80–7.21 (m, 9H, Ar-H, N-H_Diazep._ and 2 NH_2_.); MS (*m*/*z*, %): 308.10 (M^+^, 30); Anal. Calcd. for C_15_H_12_N_6_O_2_ (308.29): C, 58.44; H, 3.92; N, 27.26%. Found: C, 58.21; H, 3.65; N, 27.11%.

*8-Amino-15-methylbenzo[b]benzo[2′,3′][1,4]diazepino[6′,5′:5,6]pyrano[3,4-f][1,4]oxazepin-7(5H)-one* (**13**). Off-white powder (EtOH) with a 65% yield; mp 241–243 °C; IR (KBr): (cm^−1^) 1610–1623 (2C=N_*str*._), 1705 (C=O_*str*._), 3206–3325 (N-H_*str*._, N-H_2*str*._); ^1^H NMR (300 MHz, DMSO-*d*_6_): δ 2.01 (s, 3H, Me), 6.80–7.21 (m, 9H, Ar-H and N-H_Diazep__._), 8.61 (brs, 2H, NH_2_, D_2_O-exchangeable); MS (*m*/*z*, %): 358.11 (M^+^, 43); Anal. Calcd. for C_20_H_14_N_4_O_3_ (358.35): C, 67.03; H, 3.94; N, 15.63%. Found: C, 66.86; H, 3.67; N, 15.42%.

*8-Amino-15-methylbenzo[b]benzo[2′,3′][1,4]diazepino[6′,5′:5,6]pyrano[3,4-f][1,4]thiazepin-7(5H)-one* (**14**). Yellow crystals (MeOH/dioxane (2:1)) with a 75% yield; mp 176–178 °C; IR (KBr): (cm^−1^) 1610–1623 (2C=N_*str*._), 1705 (C=O_*str*._), 3206–3325 (N-H_*str*._, N-H_2*str*._); ^1^H NMR (300 MHz, DMSO-*d*_6_): δ 2.01 (s, 3H, Me), 6.80–7.21 (m, 9H, Ar-H and N-H_Diazep._), 8.60 (brs, 2H, NH_2_, D_2_O-exchangeable); MS (*m*/*z*, %): 374.08 (M^+^, 15); Anal. Calcd. for C_20_H_14_N_4_O_2_S (374.42): C, 64.16; H, 3.77; N, 14.96%. Found: C, 64.01; H, 3.51; N, 14.58%.

*4-((1,3-Dihydroxy-2-(hydroxymethyl)propan-2-yl)amino)-5-methyl-2-oxo-2,11-dihydrobenzo[b]pyrano[2,3-e][1,4]diazepine-3-carbonitrile* (**15**). Yellowish-brown solid (MeOH) with a 75% yield; mp 215–217 °C; IR (KBr): (cm^−1^) 1615 (C=N_*str*._), 1705 (C=O_*str*._), 2217 (C≡N_*str*._), 3226 (2N-H_*str*._), 3406 (3 O-H_*str*_); ^1^H NMR (300 MHz, DMSO-*d*_6_): δ 2.01 (s, 3H, Me), 3.20 (s, 6H, 3CH_2_), 3.61 (brs, 3H, 3 OH, D_2_O-exchangeable), 4.02 (brs, 1H, NH, D_2_O-exchangeable), 6.80–7.21 (m, 5H, Ar-H and N-H_Diazep__._); ^13^C NMR (75 MHz, DMSO-*d*_6_): 26.3 (Me), 63.4 (3CH_2_), 114.6 (C≡N), 63.5, 74.9 (4C-C), 63.3, 76.8, 113.5, 123.5, 124.1, 126.6, 128.5, 137.3, 138.1, 181.9 (10 C=C), 162.3 (C=O), 164.5 (C=N); MS (*m*/*z*, %): 370.13 (M^+^, 25); Anal. Calcd. for C_18_H_18_N_4_O_5_ (370.36): C, 58.37; H, 4.90; N, 15.13%. Found: C, 58.12; H, 4.74; N, 15.02%.

*5-Methyl-2-oxo-4-(phenylamino)-2,11-dihydrobenzo[b]pyrano[2,3-e][1,4]diazepine-3-carbonitrile* (**16**). White powder (EtOH) with an 80% yield; mp 232–234 °C; IR (KBr): (cm^−1^) 1612 (C=N_*str*._), 1705 (C=O_*str*._), 2209 (C≡N_*str*._), 3206 (2N-H_*str*._); ^1^H NMR (300 MHz, DMSO-*d*_6_): δ 2.01 (s, 3H, Me), 6.80–7.21 (m, 10H, Ar-H and N-H_Diazep__._), 8.71 (brs, 1H, NH, D_2_O-exchangeable); MS (*m*/*z*, %): 342.10 (M^+^, 10); Anal. Calcd. for C_20_H_14_N_4_O_2_ (342.35): C, 70.17; H, 4.12; N, 16.37%. Found: C, 70.01; H, 4.02; N, 16.13%.

*4-(Diethylamino)-5-methyl-2-oxo-2,11-dihydrobenzo[b]pyrano[2,3-e][1,4]diazepine-3-carbonitrile* (**17**). Light brown solid (EtOH) with a 65% yield; mp 182–184 °C; IR (KBr): (cm^−1^) 1612 (C=N_*str*._), 1705 (C=O_*str*._), 2209 (C≡N_*str*._), 3206 (N-H_*str*._); ^1^H NMR (300 MHz, DMSO-*d*_6_): δ 1.16 (t, 6H, 2CH_2_CH_3_), 2.01 (s, 3H, Me), 3.35 (q, 4H, 2CH_2_CH_3_), 6.80-7.21 (m, 5H, Ar-H and N-H_Diazep__._); MS (*m*/*z*, %): 322.14 (M^+^, 25); Anal. Calcd. for C_18_H_18_N_4_O_2_ (322.36): C, 67.07; H, 5.63; N, 17.38%. Found: C, 66.86; H, 5.35; N, 17.14%.

*5-Methyl-4-morpholino-2-oxo-2,11-dihydrobenzo[b]pyrano[2,3-e][1,4]diazepine-3-carbonitrile* (**18**). Buff solid (EtOH) with a 70% yield; mp 142–144 °C; IR (KBr): (cm^−1^) 1612 (C=N_*str*._), 1705 (C=O_*str*._), 2209 (C≡N_*str*._), 3206 (N-H_*str*._); ^1^H NMR (300 MHz, DMSO-*d*_6_): δ 2.01 (s, 3H, Me), 3.64 (t, 4H, Morpho._(C2),(C6)_-H_4_), 3.83 (m, 4H, Morpho._(C3),(C5)_-H_4_), 6.80–7.21 (m, 5H, Ar-H and N-H_Diazep__._); ^13^C NMR (75 MHz, DMSO-*d*_6_): 26.1 (Me), 52.4, 67.3 (4CH_2_), 114.6 (C≡N), 59.3, 76.7, 113.5, 123.5, 124.1, 126.6, 128.5, 137.3, 138.1, 184.9 (10 C=C), 162.3 (C=O), 164.5 (C=N); MS (*m*/*z*, %): 336.12 (M^+^, 9); Anal. Calcd. for C_18_H_16_N_4_O_3_ (336.34): C, 64.28; H, 4.79; N, 16.66%. Found C, 64.10; H, 4.43; N, 16.28%.

*5-Methyl-4-(4-methylpiperazin-1-yl)-2-oxo-2,11-dihydrobenzo[b]pyrano[2,3-e][1,4]diazepine-3-carbonitrile* (**19**). Buff solid (EtOH) with a 75% yield; mp 193–195 °C; IR (KBr): (cm^−1^) 1612 (C=N_*str*._), 1705 (C=O_*str*._), 2209 (C≡N_*str*._), 3206 (N-H_*str*._); ^1^H NMR (300 MHz, DMSO-*d*_6_): δ 2.01 (s, 3H, Me), 2.21 (s, 3H, Me), 2.32 (t, 4H, Piperaz._(C2),(C6)_-H_4_), 2.76 (t, 4H, Piperaz._(C3),(C5)_-H_4_), 6.80–7.21 (m, 5H, Ar-H and N-H_Diazep__._); MS (*m*/*z*, %): 349.15 (M^+^, 15); Anal. Calcd. for C_19_H_19_N_5_O_2_ (349.39): C, 65.32; H, 5.48; N, 20.04%. Found C, 65.11; H, 5.23; N, 19.81%.

*5-Methyl-2-oxo-4-(piperazin-1-yl)-2,11-dihydrobenzo[b]pyrano[2,3-e][1,4]diazepine-3-carbonitrile* (**20**). Off-white powder (EtOH) with an 80% yield; mp 219–221 °C; IR (KBr): (cm^−1^) 1612 (C=N_*str*._), 1705 (C=O_*str*._), 2209 (C≡N_*str*._), 3206–3211 (2N-H_*str*._); ^1^H NMR (300 MHz, DMSO-*d*_6_): δ 2.32 (t, 4H, Piperaz._(C2),(C6)_-H_4_), 2.76 (t, 4H, Piperaz._(C3),(C5)_-H_4_), 3.01 (s, 1H, N-H_Piperaz._), 6.80–7.21 (m, 5H, Ar-H and N-H_Diazep__._); MS (*m*/*z*, %): 335.10 (M^+^, 30); Anal. Calcd. for C_18_H_17_N_5_O_2_ (335.36): C, 64.47; H, 5.11; N, 20.88%. Found C, 64.25; H, 5.01; N, 20.52%.

*2-(4-Imino-13-methyl-5-oxo-5,7-dihydro-4H-*[1,3]*thiazino[4′,5′:4,5]pyrano[2,3-e]benzo[b][1,4]diazepin-2-yl) acetonitrile* (**21**). Yellow solid (MeOH/dioxane (2:1)) with an 80% yield; mp 206–208 °C; IR (KBr): (cm^−1^) 1610–1623 (2C=N_*str*._), 1705 (C=O_*str*._), 2209 (C≡N_*str*._), 3206–3325 (2N-H_*str*._); ^1^H NMR (300 MHz, DMSO-*d*_6_): δ 2.01 (s, 3H, Me), 4.15 (s, 2H, CH_2_), 6.80–7.21 (m, 9H, Ar-H and N-H_Diazep._), 8.91 (brs, H, =NH, D_2_O-exchangeable); MS (*m*/*z*, %): 349.06 (M^+^, 45); Anal. Calcd. for C_17_H_11_N_5_O_2_S (349.37): C, 58.44; H, 3.17; N, 20.05%. Found: C, 58.20; H, 3.01; N, 19.76%.

*5-Amino-14-methyl-2-phenylbenzo[b]pyrazolo[1″,5″:1′,2′]pyrimido[4′,5′:4,5]pyrano[2,3-e][1,4]diazepin-6(8H)-one* (**22**). Pale yellow crystalline solid (EtOH) with an 85% yield; mp 228–230 °C; IR (KBr): (cm^−1^) 1610–1623 (3C=N_*str*._), 1705 (C=O_*str*._), 3206–3325 (N-H_*str*._, N-H_2*str*._); ^1^H NMR (300 MHz, DMSO-*d*_6_): δ 2.01 (s, 3H, Me), 6.54 (s, 1H, Pyraz._(C4)_-H), 6.80–8.03 (m, 12H, Ar-H, N-H_Diazep._ and NH_2_.); MS (*m*/*z*, %): 408.13 (M^+^, 10); Anal. Calcd. for C_23_H_16_N_6_O_2_ (408.41): C, 67.64; H, 3.95; N, 20.58%. Found: C, 67.32; H, 3.68; N, 20.26%.

*5-Amino-14-methyl-*[1,2,4]*triazolo[1″,5″:1′,2′]pyrimido[4′,5′:4,5]pyrano[2,3-e]benzo[b]*[1,4]*diazepin-6(8H)-one* (**23**). Pale yellow powder solid (EtOH) with a 72% yield; mp 241–243 °C; IR (KBr): (cm^−1^) 1610–1623 (4C=N_*str*._), 1705 (C=O_*str*._), 3206–3325 (N-H_*str*._, N-H_2*str*._); ^1^H NMR (300 MHz, DMSO-*d*_6_): δ 2.01 (s, 3H, Me), 6.80–7.23 (m, 5H, Ar-H and N-H_Diazep._), 7.81 (brs, 2H, NH_2_, D_2_O-exchangeable), 8.65 (s, 1H, Triaz._(C3)_-H); ^13^C NMR (75 MHz, DMSO-*d*_6_): 19.8 (Me), 83.9, 107.3, 113.5, 123.5, 124.1, 126.5, 128.5, 138.2, 139.1, 168.6 (9 C=C), 150.3 (C=O), 155.2, 156.1, 164.5, 166.1 (4C=N); MS (*m*/*z*, %): 333.09 (M^+^, 55); Anal. Calcd. for C_16_H_11_N_7_O_2_ (333.30): C, 57.66; H, 3.33; N, 29.42%. Found: C, 57.39; H, 3.13; N, 29.19%.

*6-Amino-15-methyl-4-thioxo-3,4-dihydro-2H-benzo[b]pyrimido[1″,6″:1′,2′]pyrimido[4′,5′:4,5]pyrano[2,3-e][1,4]diazepine-2,7(9H)-dione* (**24**). Light brown crystalline solid (EtOH) with an 80% yield; mp 195–197 °C; IR (KBr): (cm^−1^) 1321 (C=S_*str*._), 1610–1623 (2C=N_*str*._), 1705–1715 (2C=O_*str*._), 3206–3325 (2N-H_*str*._, N-H_2*str*._); ^1^H NMR (300 MHz, DMSO-*d*_6_): δ 2.06 (s, 3H, Me), 6.80–7.21 (m, 5H, Ar-H and N-H_Diazep__._), 8.52 (brs, 2H, NH_2_, D_2_O-exchangeable), 9.64 (brs, H, NH_Pyrim_., D_2_O-exchangeable); MS (*m*/*z*, %): 392.08 (M^+^, 20); Anal. Calcd. for C_18_H_12_N_6_O_3_S (392.39): C, 55.10; H, 3.08; N, 21.42%. Found: C, 55.02; H, 3.10; N, 21.19%.

*6-Imino-15-methyl-3-thioxo-3,4,6,9-tetrahydro-1H-benzo[b]pyrimido[5″,4″:5′,6′]pyrano[4′,3′:4,5]pyrano [2,3-e][1,4]diazepine-1,7(2H)-dione* (**25**). Brown solid (EtOH) with a 75% yield; mp 165–167 °C; IR (KBr): (cm^−1^) 1321 (C=S_*str*._), 1610–1623 (2C=N_*str*._), 1705–1715 (2C=O_*str*._), 3206-3325 (4N-H_*str*._); ^1^H NMR (300 MHz, DMSO-*d*_6_): δ 2.06 (s, 3H, Me), 6.80–7.21 (m, 5H, Ar-H and N-H_Diazep._), 8.68 (brs, H, =NH, D_2_O-exchangeable), 9.64, 13.21 (brs, 2H, 2NH_Pyrim_., D_2_O-exchangeable); MS (*m*/*z*, %): 393.05 (M^+^, 60); Anal. Calcd. for C_18_H_11_N_5_O_4_S (393.38): C, 54.96; H, 2.82; N, 17.80%. Found: C, 54.65; H, 2.42; N, 17.58%.

*8-Imino-10,15-dimethyl-5H-benzo[b]pyrido[1″,2″:1′,2′]pyrimido[4′,5′:4,5]pyrano[2,3-e]*[1,4]*diazepin-7(8H)-one* (**26**). Orange powder (MeOH/DMF (3:1)) with a 75% yield; mp > 300 °C; IR (KBr): (cm^−1^) 1610–1623 (3C=N_*str*._), 1705 (C=O_*str*._), 3206–3325 (2N-H_*str*._); ^1^H NMR (300 MHz, DMSO-*d*_6_): δ 2.06, 2.75 (s, 6H, 2Me), 6.80–7.21 (m, 8H, Ar-H, N-H_Diazep__._ and Pyrid._(C3,4,5)_-3H), 8.92 (brs, H, =NH, D_2_O-exchangeable); ^13^C NMR (75 MHz, DMSO-*d*_6_): 25.2, 26.1 (2Me), 76.6, 108.2, 113.5, 115.5, 123.5, 124.1, 125.1, 126.5, 128.5, 134.1, 137.2, 138.2, 156.8, 159.6 (14 C=C), 162.7 (C=O), 150.5, 158.9, 164.5 (3C=N); MS (*m*/*z*, %): 357.12 (M^+^, 50); Anal. Calcd. for C_20_H_15_N_5_O_2_ (357.37): C, 67.22; H, 4.23; N, 19.60%. Found: C, 67.10; H, 4.11; N, 19.34%.

*8-Amino-16-methyl-7-oxo-5,7-dihydrobenzo[b]benzo[4″,5″]imidazo[1″,2″:1′,6′]pyrido[4′,3′: 4,5]pyrano[2,3-e][1,4]diazepine-15-carbonitrile* (**27**). Yellowish-brown powder (EtOH) with an 80% yield; mp 215–217 °C; IR (KBr): (cm^−1^) 1610–1623 (2C=N_*str*._), 1705 (C=O_*str*._), 2210 (C≡N_*str*._), 3206–3325 (N-H_*str*._, N-H_2*str*._); ^1^H NMR (300 MHz, DMSO-*d*_6_): δ 2.07 (s, 3H, Me), 6.51 (brs, 2H, NH_2_, D_2_O-exchangeable), 6.80–8.03 (m, 9H, Ar-H and N-H_Diazep._); MS (*m/z*, %): 406.12 (M^+^, 15); Anal. Calcd. for C_23_H_14_N_6_O_2_ (406.40): C, 67.97; H, 3.47; N, 20.68%. Found: C, 67.61; H, 3.27; N, 20.38%.

*8-Amino-16-methylbenzo[b]benzo[4″,5″]imidazo[1″,2″:1′,2′]pyrimido[4′,5′:4,5]pyrano[2,3-e]*[1,4]*diazepin-7(5H)-one* (**28**). Yellow powder (MeOH/DMF (3:1)) with a 75% yield; mp 175–177 °C; IR (KBr): (cm^−1^) 1610–1623 (3C=N_*str*._), 1705 (C=O_*str*._), 3206–3325 (N-H_*str*._, N-H_2*str*._); ^1^H NMR (300 MHz, DMSO-*d*_6_): δ 2.07 (s, 3H, Me), 6.54 (brs, 2H, NH_2_, D_2_O-exchangeable), 6.80–8.45 (m, 9H, Ar-H and N-H_Diazep._); MS (*m*/*z*, %): 382.13 (M^+^, 60); Anal. Calcd. for C_21_H_14_N_6_O_2_ (382.37): C, 65.96; H, 3.69; N, 21.98%. Found: C, 65.68; H, 3.38; N, 21.67%.

*5-Imino-14-methyl-2,3,5,8-tetrahydrobenzo[b]cyclopenta[5′,6′]pyrano[4′,3′:4,5]pyrano[2,3-e]*[1,4]*diazepin-6(1H)-one* (**31**). A solution of **5** (0.29 g, 1 mmol) and cyclopentanone (0.08 g, 1 mmol) in ethanolic piperidine solution (30 mL) was refluxed for 7 h, afterwards the reaction mixture was left to cool to rt, the obtained precipitate was filtered, dried, and recrystallized using EtOH to afford **31** as a brown solid with a 65% yield, mp 268–270 °C; IR (KBr): (cm^−1^) 1610 (C=N_*str*._), 1705 (C=O_*str*._), 3206–3325 (2N-H_*str*._); ^1^H NMR (300 MHz, DMSO-*d*_6_): δ 1.83 (m, 2H, CH_2_), 2.06 (s, 3H, Me), 2.43 (t, 2H, CH_2_), 2.81 (t, 2H, CH_2_), 6.80–7.21 (m, 5H, Ar-H and N-H_Diazep._), 8.95 (brs, H, =NH, D_2_O-exchangeable); MS (*m*/*z*, %): 333.10 (M^+^, 25); Anal. Calcd. for C_19_H_15_N_3_O_3_ (333.34): C, 68.46; H, 4.54; N, 12.61%. Found: C, 68.18; H, 4.24; N, 12.31%.

*14-Methyl-2,3-dihydrobenzo[b]cyclopenta[5′,6′]pyrano[4′,3′:4,5]pyrano[2,3-e]*[1,4]*diazepine-5,6(1H,8H)-dione* (**32**). A mixture of imino analogue **31** (0.33 g, 1 mmol), HCl_conc_. (2 mL) in EtOH (30 mL) was refluxed for 1 h. After cooling the mixture was poured onto mashed ice. A yellow precipitate was collected, washed with H_2_O carefully, dried, and recrystallized using EtOH to furnish **32** with an 85% yield, mp 238–240 °C; IR (KBr): (cm^−1^) 1610 (C=N_*str*._), 1705 (2 C=O_*str*._), 3256 (N-H_*str*._); ^1^H NMR (300 MHz, DMSO-*d*_6_): δ 1.83 (m, 2H, CH_2_), 2.06 (s, 3H, Me), 2.43 (t, 2H, CH_2_), 2.81 (t, 2H, CH_2_), 6.80–7.21 (m, 5H, Ar-H and N-H_Diazep._); ^13^C NMR (75 MHz, DMSO-*d*_6_): 26.6 (2Me), 18.6, 36.1, 36.6 (3CH_2_), 76.6, 113.5, 116.1, 118.5, 123.5, 124.1, 126.5, 128.5, 137.2, 138.1, 140.6, 170.1 (12 C=C), 162.7, 162.9 (2 C=O), 164.6 (C=N); MS (*m*/*z*, %): 334.10 (M^+^, 30); Anal. Calcd. for C_19_H_14_N_2_O_4_ (334.33): C, 68.26; H, 4.22; N, 8.38%. Found: C, 68.12; H, 4.01; N, 8.15%.

*1-Acetyl-2,13-dimethyl-4H-benzo[b]pyrano[4′,3′:4,5]pyrano[2,3-e]*[1,4]*diazepine-4,5(7H)-dione* (**33**). A mixture of compound **5** (0.29 g, 1 mmol) and acetylacetone (0.10 mL, 1 mmol) was refluxed in ethanolic-piperidine solution (25 mL) for 6 h. Upon cooling the reaction mixture an off-white precipitate was collected, and recrystallized using EtOH to give **33** with an 80% yield, mp 216–218 °C; IR (KBr): (cm^−1^) 1610 (C=N_*str*._), 1705 (2 C=O_*str*._), 3206 (N-H_*str*._); ^1^H NMR (300 MHz, DMSO-*d*_6_): δ 2.06, 2.53 (m, 6H, 2Me), 2.36 (s, 3H, COMe), 6.80–7.21 (m, 5H, Ar-H and N-H_Diazep__._); MS (*m*/*z*, %): 350.09 (M^+^, 10); Anal. Calcd. for C_19_H_14_N_2_O_5_ (350.32): C, 65.14; H, 4.03; N, 8.00%. Found: C, 65.01; H, 3.86; N, 7.78%.

*13-Methyl-1-thioxo-1,2-dihydro-*[1,3,5]*triazino[1″,2″:1′,5′]pyrazolo[3′,4′:4,5]pyrano[2,3-e]benzo[b]*[1,4] *diazepin-5(7H)-one* (**34**). A solution of α-aminocarbothiamide **9** (0.34 g, 1 mmol) and HC(OEt)_3_ (10 mL) was refluxed for 6 h. The mixture was cooled, a yellow solid was obtained, then collected by filtration, dried, and recrystallized using EtOH to give **34** with a 76% yield; mp 202–204 °C; IR (KBr): (cm^−1^) 1326 (C=S_*str*._), 1610–1623 (3C=N_*str*._), 1705 (C=O_*str*._), 3206–3325 (2N-H_*str*._); ^1^H NMR (300 MHz, DMSO-*d*_6_): δ 2.07 (s, 3H, Me), 6.80–7.95 (m, 6H, Ar-H, Triazi._(C6)_-1H, and N-H_Diazep._), 13.56 (brs, H, NH_Triazi_., D_2_O-exchangeable); MS (*m*/*z*, %): 350.05 (M^+^, 42); Anal. Calcd. for C_16_H_10_N_6_O_2_S (350.35): C, 54.85; H, 2.88; N, 23.99%. Found: C, 54.51; H, 2.49; N, 23.64%.

*3,13-Dimethyl-1-thioxo-1,2-dihydro-[1,3,5]triazino[1″,2″:1′,5′]pyrazolo[3′,4′:4,5]pyrano[2,3-e]benzo[b][1,4] diazepin-5(7H)-one* (**35**). A mixture of α-aminocarbothiamide **9** (0.68 g, 2 mmol) and acetyl chloride (2 mmol) was refluxed in benzene (30 mL) for 6 h. Upon cooling the isolated product was collected and recrystallized using benzene to afford **35** as an off-white powder, with a 70% yield; mp 182–184 °C; IR (KBr): (cm^−1^) 1326 (C=S_*str*._), 1610–1623 (3C=N_*str*._), 1705 (C=O_*str*._), 3206–3325 (2N-H_*str*._); ^1^H NMR (300 MHz, DMSO-*d*_6_): δ 2.07, 2.45 (s, 6H, 2Me), 6.80–7.95 (m, 5H, Ar-H and N-H_Diazep._), 13.32 (brs, H, NH_Triazi_., D_2_O-exchangeable); ^13^C NMR (75 MHz, DMSO-*d*_6_): 19.8, 21.2 (2Me), 83.9, 113.5, 114.4, 123.5, 124.1, 126.5, 128.5, 138.1, 139.2, 147.1 (10 C=C), 150.4 (C=O), 133.8, 154.3, 164.6 (3 C=N), 189.5 (C=S); MS (*m*/*z*, %): 364.07 (M^+^, 15); Anal. Calcd. for C_17_H_12_N_6_O_2_S (364.38): C, 56.04; H, 3.32; N, 23.06%. Found: C, 55.85; H, 3.15; N, 22.79%.

*2-(13-Methyl-5-oxo-1-thioxo-1,2,5,7-tetrahydro-[1,3,5]triazino[1″,2″:1′,5′]pyrazolo[3′,4′:4,5]pyrano[2,3-e] benzo[b][1,4]diazepin-3-yl)acetonitrile* (**36**). It was synthesized under the same conditions as those described for the synthesis of compound **33**. Yellow crystals (EtOH) with a 70% yield; mp 198–200 °C; IR (KBr): (cm^−1^) 1325 (C=S_*str*._), 1610–1623 (3C=N_*str*._), 1705 (C=O_*str*._), 2215 (C≡N_*str*._), 3206–3325 (2N-H_*str*._); ^1^H NMR (300 MHz, DMSO-*d*_6_): δ 2.07 (s, 3H, Me), 4.17 (s, 2H, CH_2_), 6.80–7.95 (m, 5H, Ar-H and N-H_Diazep._), 13.61 (brs, H, NH_Triazi_., D_2_O-exchangeable); ^13^C NMR (75 MHz, DMSO-*d*_6_): 19.8 (Me), 22.2 (CH_2_), 116.2 (C≡N), 83.9, 113.5, 114.4, 123.5, 124.1, 126.5, 128.5, 138.1, 139.2, 147.1 (10 C=C), 150.4 (C=O), 133.8, 154.3, 164.6 (3 C=N), 189.5 (C=S); MS (*m*/*z*, %): 389.05 (M^+^, 35); Anal. Calcd. for C_18_H_11_N_7_O_2_S (389.39): C, 55.52; H, 2.85; N, 25.18%. Found: C, 55.25; H, 2.63; N, 25.02%.

*3-(Chloromethyl)-13-methyl-1-thioxo-1,2-dihydro-[1,3,5]triazino[1″,2″:1′,5′]pyrazolo[3′,4′:4,5] pyrano[2,3-e]benzo[b][1,4]diazepin-5(7H)-one* (**37**). To a well-stirred solution of α-aminocarbothiamide **9** (0.68 g, 2 mmol) and Et_3_N (0.3 mL) in absolute EtOH (30 mL), ClCH_2_COCl (0.24 mL, 1 mmol) was added dropwise for 1 h at rt, and afterwards the mixture was warmed for 6 h at 60 °C. After cooling the reaction mixture and pouring onto mashed ice, a light brown solid was collected by filtration, dried, and recrystallized using EtOH, to furnish **37** with an 80% yield; mp 198–200 °C; IR (KBr): (cm^−1^) 1326 (C=S_*str*._), 1610–1623 (3C=N_*str*._), 1705 (C=O_*str*._), 3206–3325 (2N-H_*str*._); ^1^H NMR (300 MHz, DMSO-*d*_6_): δ 2.07 (s, 3H, Me), 3.29 (s, 2H, CH_2_), 6.80–7.95 (m, 5H, Ar-H and N-H_Diazep._), 13.63 (brs, H, NH_Triazi_., D_2_O-exchangeable); MS (*m*/*z*, %): 400.05 (M^+^+2, 10), 398.04 (M^+^, 32); Anal. Calcd. for C_17_H_11_ClN_6_O_2_S (398.83): C, 51.20; H, 2.78; Cl, 8.89; N, 21.07%. Found: C, 51.10; H, 2.56; Cl, 8.58; N, 20.85%.

*3-Mercapto-13-methyl-1-thioxo-1,2-dihydro-[1,3,5]triazino[1″,2″:1′,5′]pyrazolo[3′,4′:4,5]pyrano[2,3-e] benzo[b][1,4]diazepin-5(7H)-one* (**38**). CS_2_ (4 mmol) was added to solution of α-aminocarbothiamide **9** (0.68 g, 2 mmol) in EtOH (50 mL), afterwards the mixture was heated in water bath at 80 °C for 6 h. After evaporation of the solvent to one fourth of its volume, the mixture was poured into mashed ice. The formed product was collected, washed carefully with H_2_O, and recrystallized using MeOH to give **38** as a yellowish-brown powder, with a 65% yield; mp 213–215 °C; IR (KBr): (cm^−1^) 1326 (C=S_*str*._), 1610–1623 (3C=N_*str*._), 1705 (C=O_*str*._), 2453 (S-H_*str*._), 3206–3325 (2N-H_*str*._); ^1^H NMR (300 MHz, DMSO-*d*_6_): δ 2.07 (s, 3H, Me), 6.80–7.95 (m, 5H, Ar-H and N-H_Diazep._), 12.01 (s, 1H, S-H, D_2_O exchangeable), 13.41 (brs, H, NH_Triazi_., D_2_O-exchangeable); MS (*m/z*, %): 382.42 (M^+^, 35); Anal. Calcd. for C_16_H_10_N_6_O_2_S_2_ (382.42): C, 50.25; H, 2.64; N, 21.98%. Found: C, 50.14; H, 2.32; N, 21.69%.

### 3.2. Cytotoxic Assessment

#### Methodology

The preliminary cytotoxic impacts were achieved using the SRB method as previously reported [30,31].

## 4. Conclusions

The purpose of this study was to synthesize and evaluate the cytotoxic impact of some new benzo[*b*][1,4]diazepines. We synthesized 5-methyl-4-(methylthio)-2-oxo-2,11-dihydrobenzo[*b*] pyrano[2,3-*e*][1,4]diazepine-3-carbonitrile (**5**), and 3-amino-12-methyl-4-oxo-4,6-dihydro-2*H*-benzo[*b*]pyrazolo[3′,4′:4,5]pyrano[2,3-*e*][1,4]diazepine-2-carbothioamide (**9**), as a new bipod and tripod pharmacophoric architectures. The vitality of the terminal *o*-methylthionitrile as well as α-aminocarbothiamide tags were inspected in a sequence of treatments, including cyclocondensation at the annulation of new tri and/or tetrapod pharmacophoric analogues. The preliminary cytotoxicity declared that, majority of the examined compounds own momentous cytotoxic activities. Compound **9** was the most effective among the screened series with IC_50_ = 16.19 ± 1.35 and 17.16 ± 1.54 μM against HCT-116 and MCF-7, respectively. Moreover, the tested tumor cell lines were generally susceptible to conjugates **7**, **6**, **8**, **15**, **16**, **18**, **19**, and **20**, in a downward order, with IC_50_ < 60.00 μM.

## Figures and Tables

**Table 1:** Preliminary *in vitro* cytotoxicity values of some new synthesized compounds.

Compound No.	Cytotoxic Effect (IC_50_ (μM)) ± SD	
	MCF-7	HCT-116
**5**	95.26 ± 2.32	90.91 ± 3.21
**6**	19.48 ± 0.15	18.78 ± 1.02
**7**	17.54 ± 1.20	17.20 ± 1.12
**8**	22.18 ± 0.98	21.97 ± 0.76
**9**	17.16 ± 1.54	16.19 ± 1.35
**15**	24.54 ± 1.87	24.11 ± 2.13
**16**	33.54 ± 1.42	32.54 ± 1.28
**17**	82.02 ± 0.95	81.18 ± 0.32
**18**	46.76 ± 1.27	45.73 ± 0.87
**19**	56.24 ± 2.20	55.41 ± 0.35
**20**	60.00 ± 1.25	58.81 ± 1.28
**Doxorubicin**	10.34 ± 0.23	9.88 ± 0.15

Values in the range 1–20 indicate ‘very strong’, 21–40 indicate ‘strong’, 41–60 indicate ‘moderate’, and 61–100 indicate ‘very weak’. IC_50_ is the concentration of compound that is required to decrease the feasibility of the cells by 50%, compared to the non-treated control cells. MCF-7: Human breast tumor cell line; HCT-116: human colorectal carcinoma; and doxorubicin (Adriamycin): Positive control compound.
